# Different Effects of Alcohol on the Liver and the Pancreas

**DOI:** 10.1093/function/zqab008

**Published:** 2021-02-19

**Authors:** Ole H Petersen

**Affiliations:** School of Biosciences, Cardiff University, Wales, UK

## A Perspective on “Ethanol Disrupts Hormone-induced Calcium Signaling in Liver”

In this issue of *Function*, Gaspers et al.^1^ show that ethanol, in pathophysiologically relevant concentrations, can reduce the frequency and magnitude of hormone-induced Ca^2+^ oscillations and Ca^2+^ waves in the liver. The inhibitory effect of ethanol on Ca^2+^ signaling is proposed to be due to acetaldehyde formation catalyzed by alcohol dehydrogenase (ADH), because it was blocked when ADH was inhibited and enhanced when the enzyme acetaldehyde dehydrogenase was inhibited.^1^ Ethanol did not inhibit Ca^2+^ signal generation in response to uncaging of caged inositol-1,4,5-trisphosphate (IP_3_), so the inhibitory ethanol effect is not due to interference with IP_3_ receptor function, but must be the result of the demonstrated inhibition of IP_3_ formation.^1^ These effects of ethanol are likely to have functional consequences as Ca^2+^ signaling has previously been shown to be an important factor in controlling the output of glucose from the liver.^2^

The two organs most commonly damaged by alcohol are the liver and the exocrine pancreas, but the mechanisms by which alcohol causes damage seem very different. All available evidence indicates that the liver is principally affected by acetaldehyde generated in an oxidative process catalyzed by ADH,^1^ which is by far the dominant pathway for alcohol metabolism in that organ.^1^ In contrast, the destructive effect of ethanol on the exocrine pancreas is due to the nonoxidative combination of ethanol with long-chain fatty acids, generating the toxic fatty acid ethyl esters (FAEEs) that elicit massive overloading of the acinar cells with Ca^2+^.^3^ The pancreas has a particularly high capacity for FAEE production due to high concentrations of FAEE synthases^4^ and, unlike the results in the liver,^1^ alcohol-related damage to the pancreas is dramatically exacerbated by ADH inhibition.^4^ Thus, alcohol-related disease in the pancreas is associated with excessive Ca^2+^ signal generation, whereas the opposite would appear to be true for the liver (Table 1).

**Table 1.** Summary of the Effects of Alcohol (at Pathophysiologically Relevant Concentrations) and its Metabolites on Processes Related to Ca^2+^ Signaling in Liver Cells and Pancreatic Acinar Cells

	Liver cells	Pancreatic acinar cells
Direct ethanol effects	• Hardly any	• Hardly any
Effects requiring ADH action;	• Inhibits PLC	• None
some possibly mediated by acetaldehyde	• Uncouples cells	
Effects depending on combination of ethanol and fatty acids, generating FAEEs	• None known	• Opens IP_3_ receptors and CRAC channels
Acute effects of alcohol metabolites	• Inhibits Ca^2+^ signaling and prevents intercellular Ca^2+^ waves	• Ca^2+^ overloading causing uncoupling of cells
Disease induction	• Fatty liver	• Acute pancreatitis

ADH, alcohol dehydrogenase; PLC, phospholipase C; FAEE, fatty acid ethyl ester.

Ethanol also inhibits gap junctional communication between hepatocytes in the *ex vivo* perfused liver, causing a loss of spatially coordinated Ca^2+^ oscillations.^1^ This result is consistent with previous data from another group showing that ethanol inhibited the movement of Lucifer Yellow between cultured hepatocytes.^5^ In both studies,^1^^,^^5^ the uncoupling effect of ethanol was absent when ADH was inhibited.

Intercellular communication between adjacent liver cells was first described by Penn in 1966,^6^ in an electrophysiological study showing that an imposed voltage change in one cell was transmitted to adjacent cells in what appeared to be a three-dimensional network. Each hepatocyte seemed to be electrically connected to all its neighbors.^6^ A more detailed quantitative analysis of the electrotonic spread in the liver revealed a space constant of ∼390 μm.^7^ As the average diameter of rat liver cells is ∼18 μm^8^ this immediately shows that electrical current (movement of small ions) spreads easily through many cells in what is almost an infinite network (in relation to the dimensions analyzed). In stark contrast, the intercellular current spread in the exocrine pancreatic tissue is limited to ∼100 μm.^9^ This is because there are no gap junctions between acinar and duct cells. Therefore, in spite of very large areas of densely packed gap junctions between adjacent acinar cells, movements of ions and small molecules (up to MW 1000) are confined to individual acinar units.^9^ Thus, there is long-range intercellular communication in the liver, but only short-range communication in the pancreas. Nevertheless, pancreatic acinar cells and liver cells have in common that they possess large areas of gap junctions between adjacent cells, allowing easy passage of ions and small molecules from cell to cell.

Another feature common to hepatocytes and pancreatic acinar cells is that both cell types uncouple under pathological conditions. In the liver, ethanol closes gap junctions via a mechanism that is not fully understood, but requires metabolism catalyzed by ADH,^1^^,^^5^ whereas in the pancreatic acinar cells gap junctions would close due to excessively high cytosolic Ca^2+^ concentrations, and/or a reduction of the intracellular pH,^9^^,^^10^ resulting from the action of FAEEs.^3^ The precise mechanism by which ethanol causes liver cell uncoupling is still somewhat unclear. There is agreement, between both an earlier study^5^ and the paper in this issue of *Function*,^1^ that the uncoupling effect of ethanol is blocked by inhibition of ADH. This would suggest that the effect is mediated by acetaldehyde.^1^ However, the earlier data^5^ show that acetaldehyde itself failed to induce uncoupling.

The uncoupling of normally well-coupled cells, in both the liver and the pancreas under pathological conditions, is an intriguing and potentially important phenomenon, but its precise significance for the pathophysiology of alcohol-related diseases is far from clear. The basic problem is that we still, so many years after the discovery of gap junctional communication in these electrically nonexcitable tissues,^6^^,^^10^ do not fully understand the functional importance of gap junctions in the normal physiology of these two organs. Gap junctional communication allows synchronous or near-synchronous activation of multicellular assemblies, but it is only in the heart that the importance of this is completely clear. With regard to this organ, it is immediately obvious that without coordinated contraction and relaxation of all muscle cells in an atrium or a ventricle, the heart could not perform its vital pump function. It is, however, not so easy to explain why synchronous activation of many liver or pancreatic cells would necessarily be advantageous for the output of glucose or secretion and therefore it is also not clear why uncoupling of neighboring cells in these tissues, by closure of gap junctions, would cause functional problems. With regard to the liver, it has been argued that intercellular propagation of Ca^2+^ signals is a mechanism by which a relatively small number of cells, activated by a low concentration of a hormone, could recruit an entire lobule into an integrated metabolic response.^2^ This could help the lobular response to norepinephrine released from sympathetic nerve terminals.^2^ This is a plausible hypothesis worth further testing. The closure of gap junctions in the exocrine pancreas, occurring in response to an excessive rise in the cytosolic Ca^2+^ concentration, or a decrease in the intracellular pH, could be regarded as an in-built protection mechanism, whereby an injured cell would be isolated from its neighbors in order not to allow a toxic high Ca^2+^ concentration or a low intracellular pH to spread and thereby destroy many other cells.^9^^,^^10^ However, in the liver it would appear that ethanol, in pathophysiologically meaningful concentrations, does not increase the cytosolic Ca^2+^ concentration, but actually reduces Ca^2+^ signal generation.

Overall, it is remarkable how very different the actions of alcohol, and its mechanisms of action, are in the pancreas and the liver (Table 1). Further elucidation of these differences may provide helpful clues relevant to our understanding of the physiology and pathophysiology of these organs.

## Funding

The author acknowledges funding from the UK’s Medical Research Council (MR/J002771/1; G19/22).

## Conflicts of Interest Statement

None declared.
